# The utility of a new point-of-care test for synthetic cannabinoids: a mixed-methods study in people who use synthetic cannabinoids and stakeholders

**DOI:** 10.1186/s12954-025-01227-7

**Published:** 2025-06-30

**Authors:** Martine Skumlien, Sam Craft, Luke Russell, Navyaa Toshniwal, Christopher Pudney, Tom P. Freeman, Jenny Scott

**Affiliations:** 1 Addiction and Mental Health Group (AIM), Department of Psychology, University of Bath, Bath, UK; 2 Department of Addictions, King’s College London, London, UK; 3 Department of Life Sciences, University of Bath, Bath, UK; 4 Centre for Academic Primary Care, Bristol Medical School, University of Bristol, Bristol, UK

**Keywords:** Synthetic cannabinoids, Spice, Drug testing, Drug checking, Homelessness, Point of care technology

## Abstract

**Background:**

Synthetic cannabinoids (SCs) are prevalent in prisons and among people who experience homelessness in the UK and can cause serious harms to people who use them. While it is currently not possible to test for SCs at the point of care (POC), a new method for doing so using fluorescence spectral fingerprinting has recently been developed. In this study, we aimed to outline the potential utility of POC SC testing in settings where SCs and SC use occur.

**Methods:**

This is a mixed-methods study. Professional stakeholders (*n* = 449) working or volunteering in healthcare, homeless, police, or prison services were invited to give their views on POC SC testing in an online survey with both quantitative and qualitative (free text) questions. Follow-up interviews were conducted with 35 survey participants and additionally with 25 people who use synthetic cannabinoids (PWUSC). Quantitative survey responses on the overall view of saliva- or drug-based detection of SCs were compared between the four groups using Kruskal-Wallis tests. Qualitative survey responses were analysed using content analysis and interviews were analysed using thematic analysis.

**Results:**

SCs were perceived as prevalent in UK prisons and homeless communities, but stakeholders felt poorly equipped to tackle SC use and harms. The quantitative analyses revealed that all groups rated both saliva- and drug-based detection positively, but police and prison services were more positive towards both types of testing than healthcare and homeless services (all *p’s* < 0.001). The thematic and content analyses outlined several potential benefits of POC SC testing across all four settings, with the strongest support for use in prisons, particularly to reduce the ingress of SCs. Both PWUSC and professional stakeholders raised concerns that testing would be used in a punitive manner and highlighted the lack of treatment options for SC dependence and overdose as a major barrier to reducing harms.

**Conclusions:**

POC SC testing has the potential to support healthcare, homeless, police, and prison services in reducing the prevalence of SCs and improving the care of PWUSC. However, ethical application of the technology must be carefully considered to avoid causing undue harm to PWUSC, such as criminalisation and stigma.

**Supplementary Information:**

The online version contains supplementary material available at 10.1186/s12954-025-01227-7.

## Introduction

Synthetic cannabinoids (SCs), often colloquially named “spice”, are a category of novel psychoactive substances that were originally designed to mimic the effects of cannabis. However, while the acute effects of SCs may partly mirror those of cannabis, such as euphoria and relaxation, SCs also carry markedly greater risk of serious harms including addiction and withdrawal, psychosis, seizure, cardiorespiratory problems, and death [1–3]. As of 2024, 254 synthetic or semi-synthetic cannabinoids were monitored by the European Monitoring Centre for Drugs and Drug Addiction, with new variations still emerging [4]. Products are typically made by spraying dissolved synthetic cannabinoid onto dried herbal mixtures, paper, or vape liquids, which can then be vaporised or smoked in a similar way to cannabis or tobacco. This can result in considerable variability in strength within the same product, for instance, if the solution has been sprayed unevenly onto the herbal mixture or paper. This variability both within and between products means that the psychoactive effects of SCs are unpredictable, increasing the risk of harm to people who use SCs (PWUSC).

In the UK, less than 0.4% of the English and Welsh population reported past-year use of SCs in 2023 [5]. However, use is prevalent among people who experience homelessness and people living in UK prisons [6–8]. Indeed, a disproportionate number of SC-related deaths are concentrated in these groups [9], and SCs were implicated in almost half of non-natural deaths among male prisoners in England and Wales between 2015 and 2020 [10]. In addition to SC-related harms and death, PWUSC are also at risk of theft, bullying, sexual assault, and violence, sometimes due to SC-related debt or during incapacitation [11, 12], as well as stigma [13]. Moreover, although SC use is relatively low in the general population, there is increasing concern around accidental exposures through SC-contaminated vapes or edibles sold as cannabis products, especially among teenagers [14, 15]. Therefore, the issues associated with SCs cut across healthcare, homeless, and criminal legal system settings.

One key challenge to tackling these issues is the lack of point-of-care (POC) testing for SCs. POC drug testing is used in a wide range of scenarios, including to determine care pathways in healthcare settings, support adherence to addiction treatment programmes, and prevent drugs from entering prison establishments. In these situations, it is not feasible to wait for a full laboratory analysis, which is costly and can take several months to come back, before actioning a response. Similarly, addressing the problem of SCs requires assays which can rapidly identify SCs across their wide ranging and rapidly changing chemical compositions at the POC [16].

Researchers from our team have developed a new method for detecting synthetic cannabinoids in both drug materials and saliva at the POC using fluorescence spectral fingerprinting, for which proof-of-concept has been demonstrated [17] and several prototype devices have been developed [18–20]. The prototype devices are designed to detect SCs on paper and textiles and in herbal mixtures, e-cigarette liquids, and human saliva– in short, most substances in which SCs are commonly found [4, 14, 21]. The devices are ultraportable, rechargeable, easy to use, and give instant results. Results are displayed on a small screen or indicated by a panel of lights which change colour according to the result (white = negative, red = positive). To our knowledge, these are the first and only tests capable of generically detecting SCs in saliva and drug materials at the POC.

To understand the potential utility of POC SC testing, it is necessary to explore how the test might be received and used by both PWUSC and stakeholders working in relevant settings. Therefore, the aim of the current mixed-methods study was to outline the scope for POC SC testing in healthcare, homelessness, police, and prison settings through a survey and interviews with stakeholders working in these settings (hereafter referred to as “stakeholders”) and with PWUSC. The study was informed by the COM-B model for behaviour change, which suggests that interventions that aim to change a behaviour– in this case, the adoption of a new test for POC SC detection– must target the capability (C), opportunity (O), and motivational (M) factors which underpin that behaviour (B) [22]. The COM-B framework has been used to guide a range of health-related behaviours, such as interventions for smoking cessation [23, 24]. We aimed to identify:

1. The settings in which POC SC detection is likely to be useful and the impact it can have in these settings.
2. How the POC SC test can be integrated into practice in different settings, and key factors that may facilitate or act as barriers to doing so.
3. Any foreseeable unintended or unwanted consequences of implementing POC SC testing.

## Methods

The study, including aims and analyses, was pre-registered to the Open Science Framework [25]. Deviations from the protocol are outlined in the Supplemental Materials. Data were collected between March 2023 and May 2024.

### Design

This is a mixed methods study with three parts: (1) qualitative interviews with PWUSC; (2) a cross-sectional survey for stakeholders, containing both quantitative and qualitative questions; and (3) qualitative interviews with stakeholders. We used an embedded design, whereby the qualitative interviews were the primary method used to address our research questions and the survey data were used to support and contextualise insights gained from the interviews.

### Participants

Eligibility criteria for all participants were being at least 18 years old and fluent in English. PWUSC were additionally required to have lived experience of SC use but there was no requirement on minimum frequency of use. PWUSC were purposively recruited from homeless services in the Southwest of England. Service staff acted as gatekeepers and referred prospective participants to the researcher, who provided further information and took consent. All participants were given a £20 gift voucher to compensate them for their time.

Stakeholders had to have professional experience with SCs or people using SCs and work/volunteer in one of the following sectors: healthcare, homeless services, police, or prison. Stakeholders were purposively recruited through the following channels:

- The Royal College of Psychiatry and the Royal College of Emergency Medicine newsletters.
- Charity Choice, a large directory of charities in the United Kingdom. The researchers emailed all listed homeless charities on www.charitychoice.co.uk with a valid email address.
- The National Crime Agency.
- Contacts of the research team within healthcare, homeless services, police, and prison, who were asked to share the study with their networks.

Prospective stakeholder participants were sent a link to a survey about their experience with SCs. We aimed to recruit at least 180 participants for the survey (*n* = 45 in each group), based on a quantitative power calculation of the minimum number required to detect a medium effect size *f* = 0.25 for the difference between the four groups with 80% power. At the end of the survey, participants were asked if they would like to be contacted for a follow-up interview. Of those who opted in, interview participants were selected based on their general views on SC detection (positive or negative), as well as their job role, UK region, gender, and race/ethnicity. This was to ensure that a range of perspectives were captured. All stakeholder participants, except those working in His Majesty’s Prison and Probation Service (HMPPS), were offered a £10 voucher to compensate their time for completing the survey and a £20 voucher for completing the interview. HMPPS staff are not permitted to receive reimbursement for participation in research studies.

Recruitment for the qualitative PWUSC and stakeholder interviews were guided by information power [26]. For the PWUSC interviews, we had a narrow study aim and dense sample, and used established theory (i.e., the COM-B framework) in a cross-case analysis. The interviews were of varying quality and information value, and recruitment continued until enough high-quality interviews had been obtained, resulting in a sample of *n* = 25 PWUSC participants. For the stakeholder interviews, we had a broad study aim and sparse sample, and used established theory in a cross-case analysis. In this case almost all interviews were judged to have strong informational value, thus *n* = 8 in each stakeholder group was considered sufficient. The exception to this was the healthcare group, which was more diverse with respect to job role, resulting in a larger sample of *n* = 11.

Ethical approval was obtained from the University of Bath Research Ethics Committee (EP 22 024; 0316–405) and from the HMPPS National Research Committee (2023 − 303). Informed consent was taken from all participants at the start of the survey and before the interview.

### Procedures and materials

#### Survey

The survey link was distributed to potential participants via email, either directly from the researcher or through a third party (stakeholder contacts, newsletters), and completed online in QuestionPro. The survey included both quantitative/numeric response questions and qualitative/free text response questions about participants’ experience with SCs, the usefulness of the new POC device for detection of SCs in saliva and drug materials, factors that might help or hinder the implementation of the device, and demographics. The main outcomes of the survey were how positive or negative participants were towards SC detection in (1) saliva and (2) drug materials within their setting, ranked on an 11-point scale from − 5 (very negative) to 5 (very positive).

#### Interviews

The stakeholder interviews took place online via Microsoft Teams. Participants were emailed information and consent forms in advance and asked to return the signed consent form before the interview. The interview was semi-structured and followed a topic guide informed by the COM-B model for behaviour change [22]. PWUSC interviews took place in a quiet room at the service site. Staff informed service users about the study and referred interested participants to the researcher, who provided them with further information. After giving informed consent, participants were asked to complete a short demographic and drug use questionnaire followed by a semi-structured interview. The topic guide included questions about their experience of using SCs and their views on SC testing in different settings.

The interview topic guides were developed based on existing literature, input from our stakeholder partners, and team expertise. They were applied flexibly and amended to reflect new subjects that emerged during data collection. All interviews were conducted by MS, audio recorded, and transcribed verbatim with anonymisation by professional transcribers.

### Epistemology

The study was undertaken using an implicit critical realist perspective. This assumes that there is a perspective-independent and unobservable reality, but that research is confined to observable and perspective-dependent phenomena. The researchers, none of whom had lived experience of homelessness or SC use, actively considered their role in generating findings throughout the research process, for instance through reflexive memos and team discussions.

### Analyses

#### Survey

Participants’ attitudes (positive or negative) towards (1) saliva-based and (2) drug-based detection of SCs within their setting were compared between the four stakeholder groups using Kruskal-Wallis tests (due to strong negative skew) in R.4.2.0 [27]. Post hoc analyses were conducted using Bonferroni-corrected Wilcoxon Rank Sum tests. Free-text response questions were analysed using content analysis. The coding framework was based on the results of the interview thematic analysis (below) with new codes added iteratively. Coding was done by MS, LR, and NT, with double-coding of ≥ 20% of responses.

#### Interviews

Interview transcripts were analysed using reflexive thematic analysis [28] led by MS. PWUSC and stakeholder interviews were analysed using the same coding framework which incorporated both deductive and inductive codes. The deductive codes were based on the research question and the COM-B framework for behaviour change [22]. The inductive codes were based on initial coding of three PWUSC transcripts and eight stakeholder transcripts (two in each group), with second coding of four transcripts by JS. The resulting framework was agreed through consensus discussion between the core research team (MS, TPF, and JS) and used to code the remaining transcripts, with additional codes added inductively. Transcripts were coded using NVivo (NVivo Windows Release 1.7.1).

Following this, codes were reviewed, revised, and visualised in a thematic map of candidate themes and sub-themes. The thematic map was then reviewed by the core research team, who agreed on a coherent set of themes that followed a central organising concept based on the dataset and the research question. Themes that did not fit within this organising concept were separated to be reported in another paper. These mainly focused on the experiences and treatment needs of PWUSC, rather than on SC testing.

## Results

### Quantitative survey results

**Fig. 1:**
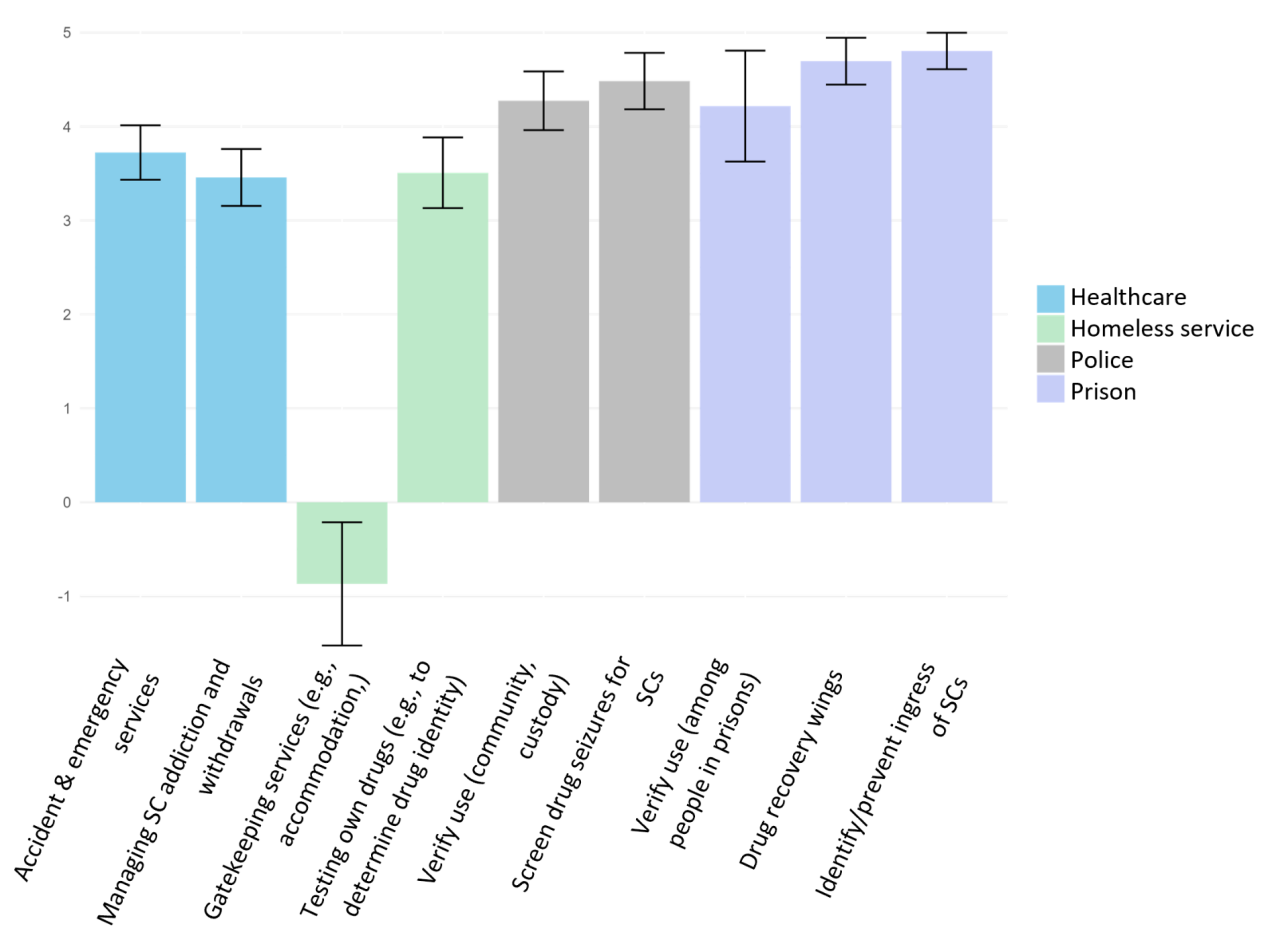
Usefulness of a new point-of-care device for detecting synthetic cannabinoids for specific purposes. Responses were rated on a scale of -5 (not at all useful) to 5 (definitely useful). Bars represent means and error bars indicate 95% confidence intervals. Abbreviations: SC, synthetic cannabinoids

A total of 449 eligible stakeholders took part in the survey. All four stakeholder groups were generally positive towards improved detection of SCs (see Table 1), although there were significant group differences in views on both saliva-based detection (*H* = 77.59, *df* = 3, *p* <.001) and drug-based detection (*H* = 88.25, *df* = 3, *p* <.001). Bonferroni-corrected post hoc pairwise Wilcoxon rank sum tests revealed that individuals working in police and prison services viewed both types of testing more favourably than those working in healthcare and homeless services (all *p’s* < 0.001). The healthcare group also viewed saliva-based detection more favourably than the homeless services group (*p* <.001). Figure 1 shows usefulness ratings of the POC detection device for different purposes.

**Table 1:** Survey study demographics

	Healthcare services (*n* = 187)	Homeless services (*n* = 145)	Police services (*n* = 67)	Prison services (*n* = 50)
**Age**, mean (sd)	38.9 (9.4), *n* = 119	38.8 (10.6), *n* = 101	41.2 (8.3), *n* = 49	42.5 (10.5), *n* = 26
**Gender**, n (%)				
Male	68 (37%)	37 (26%)	48 (72%)	20 (40%)
Female	97 (52%)	87 (60%)	11 (16%)	16 (32%)
Non-binary	1 (< 1%)	1 (< 1%)	0	0
Prefer not to say	6 (3%)	2 (1%)	2 (3%)	2 (4%)
Missing	15 (8%)	18 (12%)	6 (9%)	12 (24%)
**Race/ethnicity**, n (%)				
Asian	32 (17%)	3 (2%)	1 (< 1%)	0
Black	4 (2%)	3 (2%)	0	1 (2%)
Mixed	5 (3%)	9 (6%)	0	1 (2%)
White	117 (63%)	111 (77%)	55 (82%)	34 (68%)
Prefer not to say	14 (7%)	1 (< 1%)	5 (7%)	2 (4%)
Missing	15 (8%)	18 (12%)	6 (9%)	12 (24%)
**UK region**, n (%)				
England– North	49 (26%)	42 (29%)	9 (13%)	16 (32%)
England– Mid	29 (16%)	28 (19%)	13 (19%)	11 (22%)
England– South	73 (39%)	74 (51%)	43 (64%)	17 (34%)
Scotland	30 (16%)	1 (< 1%)	2 (3%)	0
Wales	4 (2%)	0	0	5 (10%)
Northern Ireland	2 (1%)	0	0	1 (2%)
**Years in profession**, mean (sd)	11.1 (8.7)	7.3 (6.1)	12.8 (7.9)	9.9 (10.3)
Views on SC and SC detection				
Currently has the necessary tools to meet the needs of PWUSC^a^, mean (sd)	-1.2 (2.9), *n* = 175	-0.6 (3.0), *n* = 135	-1.9 (2.8), *n* = 36	-0.8 (2.8), *n* = 48
Currently has the necessary tools to prevent import/ distribution of SCs ^a^, mean (sd)	NA	NA	-2.1 (2.6), *n* = 56	-2.1 (3.0), *n* = 47
**Overall view**^**b**^, mean (sd)				
Detection in saliva	3.8 (1.5), *n* = 173	1.9 (3.0), *n* = 128	4.6 (0.9), *n* = 62	4.4 (1.4), *n* = 42
Detection in drugs	2.7 (2.6), *n* = 173	2.2 (2.8), *n* = 128	4.6 (0.9), *n* = 62	4.9 (0.4), *n* = 42

^a^Score from − 5 (not at all) to 5 (definitely)

^b^Score from − 5 (very negative) to 5 (very positive)

Abbreviations: NA, not applicable; PWUSC, people who use synthetic cannabinoids; SC, synthetic cannabinoids, sd, standard deviation

Note: Fifteen healthcare service, 18 homeless service, six police service, and 12 prison service participants terminated the survey early and were therefore not presented with questions about age, gender, and race/ethnicity, which were included at the end

**Table 2:** Interview study demographics

	Healthcare (*n* = 11)	Homeless (*n* = 8)	Police (*n* = 8)	Prison (*n* = 8)	PWUSC (*n* = 25)
**Age**, mean (sd)	39.3 (8.6)	38.4 (8.7)	46.4 (10.4)	47.5 (9.1)	41.9 (10.2)
**Gender**, n (%)					
Male	5 (45%)	4 (50%)	7 (88%)	7 (88%)	18 (72%)
Female	6 (55%)	4 (50%)	1 (12%)	1 (12%)	7 (28%)
**Race/ethnicity**, n (%)					
Asian	2 (18%)	1 (13%)	0	0	0
Black	2 (18%)	0	0	1 (12%)	1 (4%)
Mixed	0	2 (25%)	0	0	0
White	7 (64%)	5 (63%)	8 (100%)	7 (88%)	24 (96%)
**UK region**, n (%)					
England– North	2 (18%)	2 (25%)	1 (12%)	3 (38%)	0
England– Mid	3 (27%)	0	3 (38%)	2 (25%)	0
England– South	2 (18%)	5 (62.5%)	3 (38%)	3 (38%)	25 (100%)
Scotland	4 (36%)	1 (13%)	1 (13%)	0	0
**Living situation**, n (%)	NA	NA	NA	NA	
Temporary accommodation					21 (84%)
Council House					1 (4%)
Renting from private landlord					1 (4%)
Rough sleeping					2 (8%)
**Frequency of SC use past four weeks**, n (%)	NA	NA	NA	NA	
Daily/almost daily					13 (52%)
Weekly					6 (24%)
Once or twice					1 (4%)
Never					5 (20%)

Abbreviations: PWUSC, people who use synthetic cannabinoids; SC, synthetic cannabinoids, sd, standard deviation

### Qualitative survey and interview results

Of the 449 survey respondents, 433 (96%) answered at least one free text/qualitative question and were included in the content analysis. Roughly half (*n* = 233/52%) opted into the interview study, of which 35 were invited and took part along with 25 PWUSC (Table 2). PWUSC interviews lasted between 8 and 57 min, with a mean duration of 32 (*sd* = 13) min. Stakeholder interviews lasted between 17 and 63 min, with a mean duration of 45 (*sd* = 10) minutes.

**Fig. 2:**
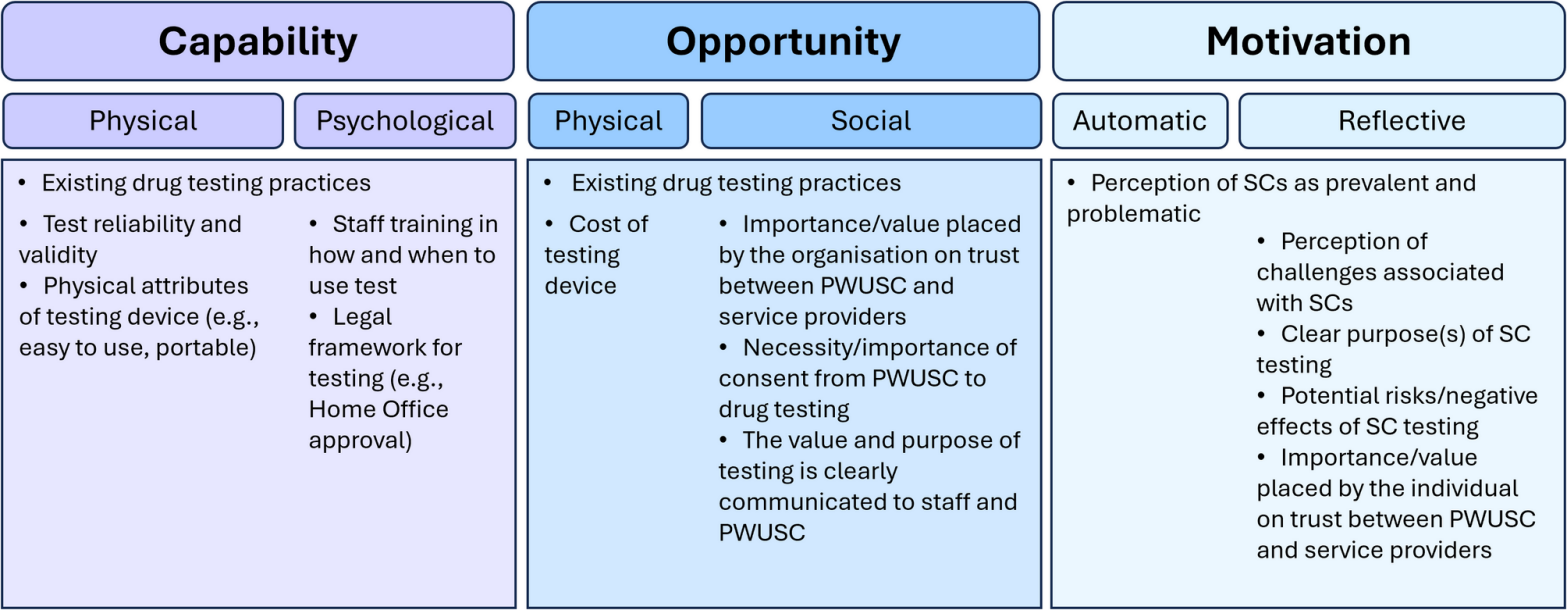
Factors important to the implementation of point-of-care testing for SCs according to the COM-B framework. Abbreviations: PWUSC, people who use synthetic cannabinoids; SC, synthetic cannabinoids

The thematic analysis produced three core themes outlining the scope for a new POC SC test in healthcare, homelessness, police, and prison settings: (1) perception of the problem of SCs (2), drug testing attitudes and capabilities, and (3) consequences of improved SC testing. The main facilitators and barriers to implementing POC SC testing, its potential areas of use, and possible harmful or unintended consequences, all based on our three main aims and contained within these themes, are outlined in supplemental Tables 3, 4, and 5, respectively. Additionally, the factors outlined in these themes and in the content analysis are conceptualised according to the COM-B framework in Fig. 2.

*Perception of the problem of SCs.* Stakeholders working within the prison and homelessness sectors generally perceived SC use as both prevalent and problematic. This was especially the case for prisons, which were described as “flooded” with SCs:

*“I mean people literally just having discarded paper that has not been smoked and is lying on the floor and nobody’s even bothered to pick it up and smoke it because there is so much of it in the prison”* (P013, male, 36y, homeless service).

The high prevalence of SCs in prison and homeless services was echoed by PWUSC:

*“I was in [prison] and there was more spice there than what I’ve seen anywhere (…) normally going to prison you get clean and you put weight on*,* and you get out and you’re a bit better for what you were before you went in. This time I came out a lot worse than what I was before I went in.”* (I018, male, 45y, PWUSC).

SCs were encountered less often by healthcare and police, likely because SC use is relatively low in the general population. However, some police stakeholders acknowledged that their inability to test for SCs might mean that they don’t have an accurate perception of the prevalence:

*“I mean*,* we could be dealing with it quite regularly and not actually know about it (…) it is almost like a vicious cycle because we are not*,* we haven’t seen it because*,* possibly because we are not screening for it*,* but because we have not seen it*,* we don’t screen for it.”* (P016, male, 40y, police).

Nonetheless, all stakeholder groups reported significant challenges related to SCs, which presented barriers to providing appropriate care of PWUSC and to stopping the distribution of SCs in prisons and the community. Chief among these were a lack of information and the constantly changing nature of SCs:

*“it’s very hard to detect*,* obviously we don’t know what the ingredients are*,* we don’t know what the effects are*,* and over the course of the last few years we’ve seen a different range of responses from men that use the drug from being very close to death to having just*,* say*,* for example slurring of their words”* (P035, male, 55y, prison).

As noted previously, the variability in the chemical composition of synthetic cannabinoids has historically been a barrier to POC testing. This was described by some stakeholders as one of the most important challenges to tackling SCs:

*“(…) it’s such a changing beast it’s hard to keep up with it. I feel like we’re playing catch up all the time.***So**,** when you say it changes**,** can you unpack that a little bit?***So*,* for instance we have the dogs trained to pick up a particular strand of it*,* within a few weeks that could change*,* and those dogs can no longer detect what’s coming in.”* (P026, male, 56y, prison).

*Drug testing attitudes and capabilities.* Unsurprisingly, a new POC SC test was seen as easier to implement in settings where drug testing is already routine practice. For instance, while testing drug materials does not normally fall within the remit of healthcare and homeless services, it could be easily integrated into existing workflows in police and prison settings:

*“Very easily I could*,* if I already had it when I’m back at work I’d literally woof it out onto the landing and have it as part of the old test that I’m already doing”* (P028, male, 43y, prison).

When it came to the potential utility of saliva-based testing, two related factors were pivotal: (1) consent to testing and (2) trust between PWUSC and stakeholder groups. First, while police and prisons can mandate drug testing in specific situations, e.g., for certain offences or as part of the mandatory drug testing programme in prisons, testing for most purposes within homelessness or healthcare settings would require consent. Many stakeholders thought this could make POC testing redundant:

*“if I ask somebody if they are using drugs and they say yes*,* well I don’t need it* [the drug test] *because just confirming they have said yes*,* I am not sure it is helpful. And if they say no (…) they are probably not then going to agree to a salivary test or engage in the psychosocial interventions (…)”* (P029, male, 39y, healthcare).

This might be particularly relevant in prisons, where a positive result could result in punitive action:

*“I am just trying to work through*,* so if it detects a synthetic cannabinoid and I am a synthetic cannabinoid user*,* why would I agree to that test knowing that in the custodial setting that is problematic?”* (P029, male, 39y, healthcare).

However, some thought consensual testing could be useful if PWUSC were engaged with the process and understood the rationale behind it:

*“if it becomes like a statutory thing*,* you have to do this once a week*,* obviously that would be disastrous. I think if it’s an opt-in and you work towards it and then it’s used as a useful tool for them*,* they’re engaged with it*,* they’ve bought into the reasoning behind it and all of that sort of thing*,* I could really see the benefit there.”* (P011, male, 46y, homeless service).

Second, the willingness of PWUSC to engage with services is contingent on trust in care providers. Several stakeholders within healthcare and homeless groups saw testing as antithetical to trust and potentially destructive to the sometimes-fragile relationship between client and care provider:

*“Now*,* you know*,* if me and you are starting out in a relationship*,* you know*,* as a friendship and I said to you did you have an apple yesterday and you said no I didn’t*,* and I tested you for that*,* how would that make you feel*,* would you feel like I trusted you? (…) there is a reason why someone lies and whether it be for a part of the lifestyle that they led or they lead to survive that is what they have done and it is more about us trying to tackle that and support somebody to say you can trust me I am not going to test you*,* I am not going to do any of that and when you want to tell the truth I am still here*,* I will be around.”* (P020, female, 40y, homeless service).

Indeed, several PWUSC reported being open and honest about their drug use and therefore found drug testing pointless or infantilising:

*“Instead of testing them why don’t you just say have you took spice and most of them would probably be honest and say yeah or no*,* don’t need to test them do you know what I mean*,* like*,* makes me feel like a criminal. (…) they can ask me and I’ll tell them*,* I’m 49 years of age*,* I’m a grown man*,* I can tell the truth*,* I really can you know*,* don’t need to test me.”* (I019, male, 49y, PWUSC).

Another challenge to trust is convincing clients that the purpose of drug testing is to help and support them, not to punish them (when that is the case). Healthcare professionals were especially worried about being seen as “arbiters of punishment” (P029, male, 39y, healthcare) and the risk of damage to the carer-patient relationship:

*“(…) what you don’t want to do when you are healthcare in prison is be seen as part of the punishing establishment. You need to be able to maintain that therapeutic alliance.”* (P029, male, 39y, healthcare).

Relatedly, a consistent concern among PWUSC was that drug testing would be used to “penalise and punish” them (I025, female, 41y, PWUSC), rather than to support them:

*“It is there for a punishment*,* what is the point of that*,* no I don’t see that as a good idea. I am going to tell you everything about me that I am struggling with for you to punish me*,* what is the point of that? (…) So*,* yeah*,* we can do what we have always done for years*,* run away with all our information and not tell you nothing”* (I025, female, 41y, PWUSC).

Still, PWUSC generally trusted that healthcare professionals act in the best interest of their patients and would therefore not use drug testing in a punitive manner. Moreover, as trust builds between clients and care providers, attitudes towards testing may also change:

*“A lot of the people that we’re working with*,* they think everyone is out to get them and are you just looking for a way to stop us being able to access what we need or to kick us out because we’re using*,* and they will be very negative about the offer of a test*,* whereas as they move through a process and they have a bit more understanding of it*,* or awareness*,* and a bit more confidence in us and their relationships*,* that they might actually see it more as a positive tool.”* (P011, male, 46y, homeless service).

Additional factors that may facilitate or prevent the implementation of POC SC testing are outlined in Supplemental Table 3.

*Consequences of improved SC testing.* Ultimately, the most important determinant of success for a SC POC test is the change it can produce in relevant settings (Supplemental Table 4). There was widespread agreement among prison stakeholders that a SC detection device would be useful to screen materials that are sometimes contaminated with SCs, such as paper-based mail, coming into prison. PWUSC who had experience of being in prison were also positive towards reducing the availability of SCs:

*“People would have to deal with it like maybe get off it and come out clean rather than coming out with a habit so. I mean my brother is in prison at the minute and I know he doesn’t smoke spice* [before going to prison] *but I know he has been smoking spice* [in prison] *cause he’s told me so that’s a perfect example*,* he’s never touched it before and now he’s probably going to come out with a bloody habit”* (I005, female, 43y, PWUSC).

Additionally, the device was seen as a useful tool for intelligence-led testing, for instance in cell searches of people suspected of being in possession of SCs. There was less certainty around the uses for saliva-based testing in the prison context, though a few stakeholders thought this could be integrated into voluntary/compact-based drug testing for people living in dedicated substance-free wings.

Among police stakeholders, a POC drug-based test was seen as useful to providing an early indication of drug identity for suspected SCs seized by police or encountered in the field, while a saliva-based test was seen as useful in custody suites and to test people suspected of driving under the influence. Most believed that the POC test was unlikely to meet the threshold for evidential use in criminal courts and that it would still be necessary to send samples off to forensic laboratories for full analysis. However, as this process is costly and time-consuming, the utility of POC detection would be to support on-the-ground decision-making and help police stay “ahead of the game” (P010, male, 37y, police), and to determine which samples to send off for further testing. Other purposes of testing mentioned by police stakeholders included in probation settings (saliva) and by the UK Border Force (drugs).

Most of the homeless service stakeholders interviewed worked in services with an open-door policy, where anyone, regardless of their substance use, were welcome. However, some suggested that screening for SC use could be useful to services where there is a clear purpose and incentive to having a drug-free environment, such as drug-free accommodation/”dry houses”. Additionally, some mentioned that SC testing could be used to help services determine how to support their clients and as a harm reduction tool. From the point of view of PWUSC, testing was seen as a potential deterrent to use, especially if there was a consequence to having a positive result:

*“Because everything should have some deterrent towards it (…) if they’re on probation or something like that and they say we’ll test you once a week for spice people will stop taking it.”* (I007, male, 55y, PWUSC).

However, others felt it would not make much of a difference:

*“Because most people I know who use spice are either*,* they are out on the streets or whatever anyway (…) if they get tested for it*,* they haven’t got no way to lose anything so no*,* I don’t think it would affect anything really unless you are under a probation order or something”* (I004, female, 55y, PWUSC).

Healthcare professionals, especially those working in prisons, said that SC testing could help determine the care pathway for acutely intoxicated patients. This was seen as particularly useful in cases where a patient is unresponsive, or where it is unclear whether their symptoms might be drug-induced or caused by an organic psychiatric illness. However, stakeholders working in emergency services did not believe that having a SC detection device in accident and emergency departments would be justified from a cost/benefit perspective, and that it should instead be targeted towards locations or populations where SCs are a particular problem. Moreover, they felt that identification had limited, if any, relevance to medical intervention in an emergency situation:

*“(…) we only do something if it’s going to change our outcome*,* if we’re going to treat their clinical symptoms anyway there may not be any benefit on doing a test that*,* whether it’s positive or not*,* we’re still going to treat the patient's symptoms”* (P019, female, 35y, healthcare).

Another potential value of saliva-based SC testing, as highlighted by both healthcare professionals and PWUSC, was to support people trying to reduce or stop their SC use. In this setting, testing was seen as a helpful tool to monitor the patients’ progress towards recovery (see Table 3). However, a major barrier to providing effective treatment of both acute intoxication and addiction, as highlighted by PWUSC and stakeholders, was the lack of a pharmacological intervention. This was also seen as limiting the value of a test:

*“If somebody was really poorly having used synthetic cannabinoids*,* I am not sure that knowing that they have used it would change the management (…) if there is ever some kind of cannabinoid blocking therapy we can use in the acute management of people that are seizing because of it then maybe*,* but we are not there”* (P029, male, 39y, healthcare).

Some healthcare stakeholders also thought that POC testing would be useful in psychiatric inpatient settings, for instance, to test patients on return from leave or to test suspicious materials that are sometimes brought into healthcare facilities.

Crucially, there were also concerns, both among PWUSC and stakeholders, that POC testing would have a number of unintended harmful outcomes (Supplemental Table 5), such as preventing access to necessary services, reducing the quality of care, or being used to target PWUSC in a punitive manner. Additionally, reducing the volume of SCs in prisons may lead to increased debt and debt-related violence, and PWUSC warned of “riots” brought on by withdrawals. Many participants also felt that improved testing would only result in more creative ways to produce, import, and use SCs, or that PWUSC would switch to other drugs.

## Discussion

### What is the setting and service need for POC testing of synthetic cannabinoids?

This is the first study to investigate the utility of POC SC testing. The qualitative analyses produced three key themes which unpack the potential usefulness of the test in healthcare, homelessness, police, and prison services:

1. *Perception of the problem of SCs*. There was widespread agreement among stakeholders on the challenges posed by SCs, including a paucity of knowledge and information, variability in drug composition and effects, and the lack of POC testing. Nonetheless, POC testing will likely be more relevant to services that work directly with populations who have high rates of SC use, such as prison and homeless services, than those who serve the general population among whom SC use is relatively low [5], such as healthcare and police.
2. *Drug testing attitudes and capabilities*. Although all stakeholder groups were generally positive towards improving their capability to detect SCs, implementing a new POC test will be more manageable for services that already test for other drugs. For instance, drug-based SC detection was seen as relatively easy to implement in police and prison settings, where it could be integrated with existing workflows, but may fall outside the remit of healthcare and homeless services. Moreover, although all service types may do saliva testing for specific purposes, stakeholders working in healthcare and homeless services stressed that testing could only be done with the explicit consent of patients/clients and should never be used in a way that risks undermining trust. Consequently, saliva-based testing in these settings must be done in partnership with PWUSC, which will likely restrict its applicability to those who are motivated to engage with services to manage, reduce, or stop their use.
3. *Consequences of improved SC detection.* The main utility of a drug-based detection device was to curtail the import and distribution of SCs. This was seen as particularly useful to prisons, which may have high concentrations of use within a confined space. Saliva-based detection was seen as having two main purposes: (i) improving the care of people using SCs, either in the case of acute exposure (e.g., to determine whether a presentation is SC-related) or to support those trying to reduce or stop their use (for instance, through contingency management); and (ii) to gatekeep dedicated drug-free spaces, such as incentivised substance-free living wings (prison) or dry houses (community). However, while stakeholders in all groups believed that saliva-based testing should be used to support PWUSC, it also had a clear punitive function in some settings. For instance, people who are identified as using SCs in prisons may face internal disciplinary procedures, which could result in a loss of “privileges” (e.g., entertainment, exercise). Similarly, refusing to provide a drug test in police custody can be a criminal offence.

Overall, the strongest support for POC SC testing was in prisons, where SC use was perceived as prevalent and harmful, existing capabilities supported both drug- and saliva-based detection, and stakeholders saw a clear and beneficial impact of testing for SCs. This was echoed by the quantitative results, which showed the strongest support for both saliva- and drug-based detection among prison and police stakeholders. Indeed, both qualitative and quantitative results suggested that the strongest utility of a POC device was to reduce the ingress of SCs into prisons. An ongoing trial is exploring this further in participating jails and prisons in the UK and USA.

### Support, not punish

Cutting across the three main themes of the qualitative analyses was the view that any new SC detection technology should only be used to support PWUSC. For instance, one of the main sources of scepticism towards SC testing among PWUSC, echoed by many stakeholders, was that this would be used as a way for the criminal legal system to target and punish them. They were more accepting of drug testing by healthcare, who they believed were more likely to use testing to aid treatment, rather than in a punitive manner.

Similarly, PWUSC and stakeholders were generally positive towards the use of SC testing as a harm reduction tool, for instance, to provide drug checking for SCs. Drug checking services offer chemical analysis of samples to inform clients about the identity of the drugs they plan to use, often delivered in conjunction with harm reduction advice, and have grown globally in recent years [29, 30]. In the UK, drug checking is offered by the Welsh Emerging Drugs and Identification of Novel Substances (WEDINOS) postal-based service and by the Loop at festivals and a monthly city centre-based service in Bristol, and is being explored by the City of Edinburgh Council in Scotland [31]. A large proportion of the harms of SCs stem from their variable chemical composition and potency [21, 32, 33], and drug checking therefore has the potential to reduce the risk of harmful and sometimes lethal effects from SCs. However, existing services do not currently have the capabilities to test for SCs or for other novel psychoactive substances such as novel non-prescribed benzodiazepines (‘street’ benzos) and synthetic opioids, which are causing a rise in fatalities in the UK and worldwide [34, 35]. Therefore, the current POC device is being developed to detect a wide range of novel psychoactive substances, including ‘street’ benzos and synthetic opioids, and has been trialled in drug checking services in Norway and New Zealand. However, we were unable to recruit people working in drug checking services for the current study and therefore suggest this as a potential avenue for future research.

An important factor limiting the utility of SC detection is the lack of pharmacological interventions against overdoses and withdrawals. Without this, better detection was seen as having limited capacity to improve outcomes for PWUSC. In fact, some healthcare stakeholders worried that testing could lead to *worse* care of PWUSC, as they may be less likely to receive appropriate treatment if symptoms are deemed to be “just” drug-induced. Indeed, there is previous research showing that prejudice and stigma contribute to worse care of people who use drugs [36]. Furthermore, better treatment and support of PWUSC, both pharmacological and psychosocial, is important to preventing possible harmful effects of reduced availability. For instance, many PWUSC worried that removing SCs from prisons would cause people to “riot” due to withdrawals [3, 37] and losing an important crutch which helps them cope with the prison environment [12]. Another possibility is that PWUSC would simply switch to other, potentially more dangerous drugs in the absence of SCs. Therefore, improved detection must be accompanied by both adequate treatment and measures that address vulnerability factors such as homelessness, lack of purposeful activity (in prisons), health issues, and trauma.

### Strengths and limitations

The current study is the first to explore the utility of POC testing for SCs and conveys the perspectives of stakeholders working in a wide range of settings as well as PWUSCs. The mixed methods approach allowed us to capture the views from a large number of stakeholders whilst exploring important factors supporting or limiting POC SC testing in depth. One limitation of our study is potential sample bias towards people who are interested in SC testing. For instance, it is possible that participants who chose to take part did so because they perceive SCs to be a problem, and that the current results thus represent the views of those who are particularly positively inclined towards SC testing. However, there were opposing views represented in all stakeholder groups, and participants expressing both positive and negative views towards SC testing in the survey were recruited for the interviews, where possible. Secondly, there were some settings suggested by stakeholders as suitable for SC detection which were not represented by our interview stakeholder groups, such as dry houses, community-based drug and alcohol services, and drug checking services. Therefore, follow-up research with these groups is needed to support our suggestions regarding SC testing in these settings.

## Conclusions

SC use is prevalent and causes significant harm among people who experience homelessness and people living in prisons in the UK. Services working with these populations currently have few tools to target SCs, including the ability to detect SCs at the POC. This study outlines how a novel POC synthetic cannabinoid detection device, the first of its kind, could support healthcare, homeless, police, and prison services in reducing the prevalence of SCs and improving care of PWUSC. While a detection device has utility across all four settings, it may be particularly well-suited to reducing the volume of SCs coming into UK prisons, as these are confined environments with high concentrations of SC use, where drug testing is already part of existing workflows.

Importantly, many stakeholders and PWUSC cautioned against using SC testing in a punitive manner and argued that its chief purpose should be to improve the care PWUSC. Therefore, ethical application of the device needs careful consideration, and detection must be offered alongside improved treatment options.

## Electronic supplementary material

Below is the link to the electronic supplementary material.

Supplementary Material 1

## Data Availability

The topic guides, thematic analysis coding framework, anonymised survey data, and anonymised stakeholder interview transcripts are available on the Open Science Framework (https://osf.io/3z6ja/files/osfstorage?view_only=). One transcript is not shared, as the participant’s job role prevented sufficient anonymisation. The service user interview data are not available due to the highly sensitive nature of their data and the narrow proximity of their recruitment.

## Abbreviations

HMPPS: His Majesty’s Prison and Probation Service
POC: Point-of-care
PWUSC: People who use synthetic cannabinoids
SC: Synthetic cannabinoids
UK: United Kingdom
USA: United States of America
