# Supplementary material for: The utility of a new point-of-care test for synthetic cannabinoids: a mixed-methods study in people who use synthetic cannabinoids and stakeholders
Source: Harm Reduct J. 2025 Jun 30;22:113. doi: 10.1186/s12954-025-01227-7 (PMC12207801; doi:10.1186/s12954-025-01227-7)
Supplement: Supplementary file 1 — Supplementary Material 1 [file 12954_2025_1227_MOESM1_ESM.docx]

**SUPPLEMENTAL MATERIALS**

**Title:** The utility of a new point-of-care test for synthetic cannabinoids: a mixed-methods study in people who use synthetic cannabinoids and stakeholders

**Authors:** Martine Skumlien PhD^1,2^, Sam Craft PhD^1^, Luke Russell^1^, Navyaa Toshniwal^1^, Christopher Pudney PhD^3^, Tom P Freeman PhD^1^, Jenny Scott PhD^4^

**Affiliations:**

^1^Addiction and Mental Health Group (AIM), Department of Psychology, University of Bath, Bath, UK

^2^Department of Addictions, King’s College London, London, UK

^3^Department of Life Sciences, University of Bath, Bath, UK

^4^Centre for Academic Primary Care, Bristol Medical School, University of Bristol, Bristol, UK

### **Deviations from the pre-registered study protocol**

We initially combined police and prison stakeholders into one ‘criminal legal system’ group and additionally sought to include participants working in drug checking services. However, we decided to drop the drug checking group due to a lack of responses and split the criminal legal system group into separate police and prison groups.

### **Supplemental Tables**

**Table 3.** Facilitators and barriers to implementing a new point-of-care device for detecting synthetic cannabinoids in healthcare, homelessness, police, and prison settings.

| **Facilitator or barrier** | **Setting** | **Survey n (%)** | **Evidence** |
| --- | --- | --- | --- |
| Trust between service users and providers | All groups | 16 (3.7%) | *“People may be reluctant to utilise the technology for fear that information may be used by police, probation/criminal justice social work, prison service etc.”* (survey respondent, male, 36y, homeless service) |
|  | Healthcare | 2 (1.1%) |  |
|  | Homeless | 14 (10.1%) |  |
|  | Police | 0 |  |
|  | Prison | 0 |  |
| Client/patient consent to testing | All groups | 152 (35.1%) | *“Person refusing to consent to the test or provide a sample”* (survey respondent, female, age missing, healthcare)  *“Main issue tends to be around refusal from patients which is already the case for other drug tests.”* (survey respondent, female, 33y, healthcare) |
|  | Healthcare | 73 (40.1%) |  |
|  | Homeless | 61 (43.9%) |  |
|  | Police | 8 (12.5%) |  |
|  | Prison | 10 (20.8%) |  |
| Cost of device | All groups | 147 (33.9%) | *“NHS budget and funding”* (survey respondent, female, unknown age, healthcare)  *“Potentially cost of the equipment vs benefit.”* (survey respondent, female, 33y, healthcare) |
|  | Healthcare | 91 (50.0%) |  |
|  | Homeless | 17 (12.2%) |  |
|  | Police | 28 (43.8%) |  |
|  | Prison | 11 (22.9%) |  |
| Reliability and validity of test | All groups | 78 (18.0%) | *“False positives would be potentially very problematic in acceptance of the device.”* (survey respondent, male, 37y, healthcare) |
|  | Healthcare | 130 (71.4%) |  |
|  | Homeless | 3 (2.2%) |  |
|  | Police | 14 (21.9%) |  |
|  | Prison | 9 (18.8%) |  |
| Device attributes | All groups | 79 (18.2%) | *“Easy to use, small devices which can be taken to community events, ease of storage of information, ability to use quickly without creating contamination of next specimen, data pull through.”* (survey respondent, male, 45y, healthcare) *“It would need to be cop proof, police officer[s] break everything!!”* (survey respondent, male, unknown age, police) |
|  | Healthcare | 51 (28.0%) |  |
|  | Homeless | 9 (6.5%) |  |
|  | Police | 17 (26.6%) |  |
|  | Prison | 2 (4.2%) |  |
| Training in use of the device | All groups | 158 (36.5%) | *“Professional knowledge and education would seem most key.”* (survey respondent, female, 32y, healthcare) |
|  | Healthcare | 84 (46.2%) |  |
|  | Homeless | 24 (18.0%) |  |
|  | Police | 30 (46.9%) |  |
|  | Prison | 20 (41.7%) |  |
| Clear communication on the purpose of testing | All groups | 75 (17.3%) | *“Understanding and awareness of synthetic cannabinols amongst staff. Clarity for staff that the technology is for the detection of synthetic cannabino[ids] only.”* (survey respondent, female, unknown age, healthcare)  *“Clear explanation of purpose to client”* (survey respondent, female, 54y, healthcare) |
|  | Healthcare | 20 (11.0%) |  |
|  | Homeless | 43 (30.9%) |  |
|  | Police | 3 (4.7%) |  |
|  | Prison | 9 (18.8%) |  |
| Home Office approval | All groups | 11 (2.5%) | *“As mentioned previously, the initial barrier would be ensuring that the technology was approved (by the Home Office) to allow for charges to be authorised without a full laboratory submission.”* (survey respondent, unknown gender and age, healthcare) |
|  | Healthcare | 0 |  |
|  | Homeless | 0 |  |
|  | Police | 11 (17.2%) |  |
|  | Prison | 0 |  |

Abbreviations: NHS, National Health Service.

*Note.* Results are based on thematic analysis of interview transcripts from 60 participants (n=11 healthcare, n=8 homeless service, n=8 police, n=8 prison, n=25 PWUSC) and content analysis of survey responses from 433 participants (n=182 healthcare, n=139 homeless service, n=64 police, n=48 prison).

**Table 4.** Potential uses for a new point-of-care device for detecting synthetic cannabinoids in healthcare, homelessness, police, and prison settings.

| **Purpose** | **Setting** | **Survey n (%)** | **Evidence** |
| --- | --- | --- | --- |
| Medical care of acutely intoxicated patients (saliva-based) | Healthcare | 121 (66.5%) | *“(…) we can have people who are hallucinating and seeing things and stuff like that and you want to know is this due to drugs or is this a sort of an organic or just a functional mental health issue (…) if you know drugs are involved you can be like ok we can give it a little longer while watching the person because we know that this drug will wear off at some point in time, we don’t need to sort of go in heavy handed and start with medication, because medication has its own side effects”* (P008, female, 38y, healthcare)  *“(…) I’ve been sent to hospital so many times where I’ve had a spice attack and the ambulance has just scooped me up and took me to hospital and they don’t know what’s wrong, you know what I mean, so yeah, a test would be good. I have to actually tell them.* ***And do you tell them?*** *Yeah, I have told them in the past when I woke up in hospital two days later and wondering what the hell is going on”* (I018, male, 45y, PWUSC)  *“(…) the cost/benefits would be pretty negligible (…) In the place where I work it [synthetic cannabinoid use] is not that common, they see it once or twice a month but if, if this was something which was happening on a daily basis then yeah probably, it would be really valuable.”* (P024, male, 28y, healthcare) |
| Differentiating SC use from psychiatric diagnoses (saliva-based) | Healthcare | 52 (28.6%) |  |
| Psychiatric inpatient settings (saliva-based) | Healthcare | 34 (18.7%) | *“Patients that leave the ward to go on unescorted leave and return to the ward in a state dissimilar to when they left it is a good screening tool to be able to discern if they have taken spice and this is the reason for their deterioration in mental state.”* (survey respondent, male, 29y, healthcare)  *“Mental health inpatients, to detect what is being taken and if the patient is being truthful. To detect what may be being passed around on the wards. To enforce trusts zero drugs policy.”* (survey respondent, female, 36y, healthcare) |
| Preventing SCs in healthcare facilities (drug-based) | Healthcare | 48 (26.4%) | *“Potentially help with detection when searching property or things brought into an inpatient ward.”* (survey respondent, female, 33y, healthcare) |
| Support people who want to stop using SCs | Healthcare, homeless services | 44 (13.7%) | *“(…) we don’t expect them to go cold turkey, we do expect them to sort of wean their way off and this is where a device like that would be really helpful because it would allow us to see where they are at and constantly monitor their progress along the way. (…) over time as they are working towards you know coming off it, there will be times when they get a negative drugs test and everything is fine, the reaction that they get is extremely positive because even though they know they haven’t taken anything, for them to then produce a negative drugs test is like a trophy or an award”* (P003, male, 31y, homeless service) |
| General/other support (saliva-based) | Homeless | 22 (15.8%) | *“(…) It would also help when trying to gauge the extent of someone'[s] spice use and the impact on other areas of their lives, e.g., finances, self-care etc. I think having this info to hand could aid with liaising with substance use support agencies.”* (survey respondent, male, 49y, homeless service) |
| Gatekeeping (saliva-based) | Homeless | 14 (10.1%) | *“I think within the sort of drug and alcohol pathway they would need, the reason they need to demonstrate that they are reducing their use or stopping their use is they will be eventually placed into a house in which other people are abstinent, and if they are continuing to be users that is a big problem really. So actually, then being able to demonstrate their abstinence or whatever is required I think it would be beneficial in that setting.”* (P011, male, 46y, homeless service) |
| Harm reduction and drug checking (drug-based) | Homeless | 49 (35.3%) | *“it would be very helpful to have that information and to be able to sit down and chat with somebody about something that they’ve maybe have already used or about to use, or if they have in their possession, if you are able to check that and then kind of protect somebody from potential harm that would be a great tool to have, something that obviously isn’t available at the moment.”* (P013, male, 36y, homeless service)  *“Any testing (once legal) of drugs could help users make more informed choices whether homeless or not - seems like a harm reduction no-brainer to know what you are about to use.”* (survey respondent, male, 57y, homeless service) |
| Testing people in police custody (saliva-based) | Police | 39 (60.9%) | *“I can’t see that they would, many forces would consider for the dip testing purpose having an additional second device just solely for SCRAs because I don’t think there would be the call for it.”* (P016, male, 40y, police)  *“(…) it’s kind of multiple knowledge really, it’s a confirmation of this offence has gone with this drug use again and (…) it enables you to ask the question we know that you’ve taken this, when did you take it and how much did you take and what effect does it have on you (…) we have to consider are you fit to detain, are you fit to interview, what do we need to do to take care of you on a 30-minute or 15-minute basis or whatever it might be.”* (P031, male, 54y, police) |
| Driving under the influence (saliva-based) | Police | 16 (25.0%) | *“(…) if we are correct on the assumption that SCRAs* [synthetic cannabinoid receptor agonists – another term for SCs] *will be introduced to the cannabis market, there’s a lot of people who are using cannabis and using cannabis products who are driving who are being apprehended, then it stands to reason there will be people who are using what they think to be cannabis products that could have synthetic cannabinoids in them but obviously that wouldn’t necessarily indicate on any of the samples we currently have”* (P010, male, 37y, police) |
| Pre-screening drug seizures (drug-based) | Police | 22 (34.4%) | *“(…) from our point of view or from a police point of view it would help us to know what we are sending off to a lab and what we are not. So, if we had a letter for instance that the prison were investigating and said we think this might be a synthetic cannabinoid and we could screen that and go yes it does or no it doesn’t, you know that would save us potentially a lab submission.”* (P016, male, 40y, police)  *“And from a perspective as well just like seizures if we’re looking at unidentified powders then you have the ability to sort of say well then just another tool in the armoury to say yeah that’s a SCRA and if it is then it will be from intel perspective as well you start having more awareness of stuff that’s in circulation at the time it’s in circulation, so you’re kind of ahead of the game as oppose to not like I said not seeing it until 6 months/8 months later until it’s a case and then you’re like oh we’re already behind the curve on this one because it’s been out there for this long”* (P010, male, 37y, police) |
| Field testing (drug-based) | Police | 12 (18.8%) | *“(…) if they had your portable device and they came across x y z and they were to test it, that would help them determine shall we seize shall we not seize, we don’t want to seize commodities where we don’t need to, that’s extra work for everybody”* (P023, male, 69y, police)  *“(…) you might end up detecting more spice jobs if you had a test kit to allow especially officers on the street, they’re stopping cars all the time, if they stopped a car and something was a bit weird and they couldn’t quite put their finger on it, they might just let them go, but if they had a tool to detect it and it was quite quick and easy to do, then you might find more detection of spice in the community.”* (P018, male, 43y, police) |
| Prevent SCs in custody suites (drug-based) | Police | 5 (7.8%) | *“In Police custody property can be checked before allowing it into the cell, or prior to leaving custody on bail if conditions are met.”* (survey respondent, female, 39y, police) |
| Preventing SCs from crossing the border (drug-based) | Police | 4 (6.3%) | *“UK border force detection and importation offences”* (survey respondent, male, 42y, police) |
| Probation services (saliva-based) | Police & prison | 9 (8.0%) | *“(…) It could be used to check conditions are being met with bail returns, or with probation services.”* (survey respondent, female, 39y, police) |
| Compact-based saliva testing, e.g., in ISFL or drug recovery wings (saliva-based) | Prison | 8 (16.7%) | *“So for me it would absolutely be a godsend and especially on the unit that I work on because we do do a, it’s like a compact-based drug testing, so the prisoners on my unit actually get tested twice a month due to the fact that they are on the drugs wing, they’re supposed to be abstaining so they’re aware that they will get tested, however, they are also very aware that we can’t actually test for spice. So yeah, this would literally be a godsend”* (P028, male, 43y, prison) |
| Prevent import of SCs into prison (drug-based) | Prison | 21 (43.8%) | *“I think if it can work then it’ll be kind of a revelation for us (…) I think it would help with the mail room because we would be able to quickly test a large volume of paper, also in reception so where they first arrive in prison, we are then going through X-raying their property”* (P030, male, 39y, prison)  *“That one for the paper would be very good, they’d use that in prison just go over the letters and these people be able to get their mail, because you don’t get your mail in prison. Say if you were in prison and you got kids and they drew you a picture, that gets sent to the prison and get photocopied, you’ll get a photocopy which is a white photocopy and the original would have been destroyed, which your kids took ages to do and, do you know what I mean, if they tested that and they know it’s safe, it can go in.”* (I018, male, 45y, PWUSC) |
| Field testing/ intelligence-led testing (drug-based) | Prison | 14 (29.2%) | *“(…) you can get quite a lot of synthetic cannabinoids onto say a sock, so we are seeing a sock being you know, people wanting you know over a thousand pounds for a sock based on that then what do we do. And then what, we are kind of at a loss as to, we are searching through a cell, it is going to have socks in it, isn’t it, so we don’t know which one it is, we are not going to send away every sock that we find for testing, we are not going to send away every piece of paper”* (P030, male, 39y, prison)  *“Well anytime we have a suspicion so when we find somebody that is under the influence we’ll take if there is anything that looks suspicious away, so if there is a small piece of paper that looks like it’s been torn out of something bigger, we’ll take that away or cell searches, you know, there’s a, security department collect a huge amount of evidence, sorry, intelligence and then based on that will go and do cell searches, so if it’s something handheld that is transportable we could use it you have a cell search, you’re not confident, you can run it over the paperwork in the cell (…) the uses would be almost endless.”* (P032, male, 33y, prison) |
| Intelligence-gathering (saliva- and drug-based) | All groups | 35 (8.1%) | *“(…) if we tested drugs, for example like a batch of spice, and it was linked to an individual elsewhere and it is the same, exactly the same type of spice, then you know, for us, it helps us build an intelligence picture of who has committed the crime where, who is involved”* (P017, male, 42y, police)  *“(…) it's going to be much more intelligence gathering, I think. Drug trends, even internationally, how are things passed through and how people are processing substances, and these testing systems can help understand that and then help us predict how we should respond as addiction services”* (P006, male, 43y, healthcare) |
|  | Healthcare | 8 (4.4%) |  |
|  | Homeless | 10 (7.2%) |  |
|  | Police | 10 (15.6%) |  |
|  | Prison | 7 (14.6%) |  |

Abbreviations: ISFL, incentivised substance-free living; PWUSC, people who use synthetic cannabinoids; SC, synthetic cannabinoid; SCRA, synthetic cannabinoid receptor agonist.

*Note.* Results are based on thematic analysis of interview transcripts from 60 participants (n=11 healthcare, n=8 homeless service, n=8 police, n=8 prison, n=25 PWUSC) and content analysis of survey responses from 433 participants (n=182 healthcare, n=139 homeless service, n=64 police, n=48 prison).

**Table 5.** Potential unintended or harmful consequences of point-of-care synthetic cannabinoid testing.

| **Consequence** | **Setting** | **Survey n (%)** | **Evidence** |
| --- | --- | --- | --- |
| Barrier to service access | All groups | 59 (13.6%) | *“Criminalisation of those using spice. Potential stigma or exclusion from services once confirmed someone is using spice. People no longer wanting to access services for other support if this is included.”* (survey respondent, female, 27y, homeless service)  *“In prison setting if patients know that we [prison healthcare] test for spice they may avoid seeing us due to the potentially punitive element and if/how this information is shared with the prison would need consideration.”* (survey respondent, male, unknown age, healthcare) |
|  | Healthcare | 13 (7.1%) |  |
|  | Homeless | 46 (33.1%) |  |
|  | Police | 0 |  |
|  | Prison | 0 |  |
| Distrust/friction between service users and staff | All groups | 28 (6.5%) | *“Reduce honesty from users, increase secretiveness and limit the identification of those using - this would prevent appropriate help being advise or signposted.”* (survey respondent, female, unknown age, homeless service)  *“I would be concerned that patients may feel offended if denying use of these substances and then subsequently asked to give a saliva sample - may promote distrust.”* (survey respondent, male, 28y, healthcare) |
|  | Healthcare | 10 (5.5%) |  |
|  | Homeless | 15 (10.8%) |  |
|  | Police | 1 (1.6%) |  |
|  | Prison | 2 (4.2%) |  |
| Negative impact on healthcare | All groups | 21 (4.8%) | *“The thing that was going through my head initially just, again with me being a cardigan-wearing left-wing psychiatrist, is again the punitive aspects of that from other non-addiction specialists, so if people get a positive spice reading so to speak, will there be punitive care and treatment decisions made because of that. I hear enough times with other substances, even with my general mental health colleagues, psychiatrist colleagues you know, this is “just drug-induced psychosis” when they’ve been admitted for mental health bed, it's still psychosis is my retort to that”* (P006, male, 43y, healthcare) |
|  | Healthcare | 16 (8.8%) |  |
|  | Homeless | 3 (2.2%) |  |
|  | Police | 1 (1.6%) |  |
|  | Prison | 1 (2.1%) |  |
| Promote measures to evade detection | All groups | 21 (4.8%) | *“You are just going to be more crafty with it, aren’t you.* ***You will just try to find ways around it.*** *More sneaky about it, that’s all.* ***I see.*** *More deceitful”* (I025, female, 41y, PWUSC)  *“It will drive innovation in the development of other psychoactive substances.”* (survey respondent, unknown gender and age, healthcare) |
|  | Healthcare | 7 (3.8%) |  |
|  | Homeless | 3 (2.2%) |  |
|  | Police | 5 (7.8%) |  |
|  | Prison | 6 (12.5%) |  |
| Punish/target PWUSC | All groups | 18 (4.2%) | *“If staff use the screening as a way of making someone’s life more difficult in a confined area like the prison and how do you mitigate that risk. (…) there is the unintended consequences of association with police, if it’s used in a negative way to either persecute people or get more people in the cells or kind of arrest people just so that they can be put under the mental health section.”* (P004, male, 45y, healthcare) |
|  | Healthcare | 7 (3.8%) |  |
|  | Homeless | 9 (6.5%) |  |
|  | Police | 1 (1.6%) |  |
|  | Prison | 1 (2.1%) |  |
| Stigma | All groups | 13 (3.0%) | “Staff being judgemental with patients when a positive result is obtained.” (survey respondent, male, 57y, healthcare) |
|  | Healthcare | 6 (3.3%) |  |
|  | Homeless | 7 (5.0%) |  |
|  | Police | 0 |  |
|  | Prison | 0 |  |
| Switch to other drugs | All groups | 13 (3.0%) | *“(…) so for example, when I haven’t been able to get spice or whatever, reality is I have then got resort to go and buying heroin because that is the only thing that will make me feel kind of better physically”* (I025, female, 41y, PWUSC)  *“Try and find another drug, do you know what I mean, to fill that void of the spice, go onto something different and then become addicted to that whether it’s physical or mentally.”* (I011, male, 38y, PWUSC) |
|  | Healthcare | 8 (4.4%) |  |
|  | Homeless | 2 (1.4%) |  |
|  | Police | 1 (1.6%) |  |
|  | Prison | 2 (4.2%) |  |
| Increase burden on services | All groups | 10 (2.3%) | *“I suppose it is like turning over the stone isn’t it, the more you detect then the more pressure it puts on already stretched resources. The more we know about, the more then law enforcement and others then have to deal with.”* (P025, female, 45y, police) |
|  | Healthcare | 3 (1.6%) |  |
|  | Homeless | 2 (1.4%) |  |
|  | Police | 5 (7.8%) |  |
|  | Prison | 0 |  |
| Increased debt and violence | All groups | 2 (0.5%) | *“(…) if we are able to detect drugs then the price is driven up because they have become more valuable and when the price is driven up, the debt increases and then the debt increase can sometimes result in increased levels of violence. Umm, but it is a consequence of being able to prove that somebody has done something or got something or in possession of something illegal and eventually it will even itself out.”* (P027, female, 45y, prison)  *“Do it because it will protect us, it would protect um but expect to have a riot on your hands (…) I mean there would be blood spilt, that is how bad it can be, there would be blood spilt – I mean you can get really nasty if you don’t have it, you can get nasty.”* (I002, female, 29y, PWUSC) |
|  | Healthcare | 0 |  |
|  | Homeless | 0 |  |
|  | Police | 0 |  |
|  | Prison | 2 (4.2%) |  |

Abbreviations: PWUSC, people who use synthetic cannabinoids**.**

*Note.* Results are based on thematic analysis of interview transcripts from 60 participants (n=11 healthcare, n=8 homeless service, n=8 police, n=8 prison, n=25 PWUSC) and content analysis of survey responses from 433 participants (n=182 healthcare, n=139 homeless service, n=64 police, n=48 prison).
